# Enhancement of Alkali Resistance of Glass Fibers via In Situ Modification of Manganese-Based Nanomaterials

**DOI:** 10.3390/ma16165663

**Published:** 2023-08-17

**Authors:** Guangzhou Wang, Jinzhuo Zhang, Fuxin Li, Kangli Li, Minglian Xin, Jiang Zhu, Xiaolei Lu, Xin Cheng, Lina Zhang

**Affiliations:** 1 China United Cement Pingyi, Co., Ltd., Linyi 276000, China; cucc234@163.com (G.W.); z2009jz@163.com (J.Z.); 18853962680@163.com (F.L.); 2 Shandong Provincial Key Laboratory of Preparation and Measurement of Building Materials, University of Jinan, Jinan 250022, China; lkl5552022@163.com (K.L.); 15552502962@163.com (M.X.); mse_zhuj@ujn.edu.cn (J.Z.); mse_luxl@ujn.edu.cn (X.L.); chengxin@ujn.edu.cn (X.C.)

**Keywords:** glass fibers, alkali resistance, surface modification, nanoparticles

## Abstract

Glass fibers are widely used in cement-based precast products, given the reinforcing requirements for toughness and strength. However, inferior alkali resistance hinders the effectiveness of glass fibers in reinforcing cement-based materials. In this paper, nanoparticle coatings were applied on the surface of alkali-resistant glass fiber (ARGF) as a protective layer via the in situ chemical reaction of oleic acid (OA) and potassium permanganate (PP). The morphology and constituents of the as-prepared ARGFs were examined using scanning electron microscopy (SEM) and obtaining X-ray photoelectron spectroscopy (XPS) measurements. Mass loss and strength retention were investigated to characterize alkali resistance of modified ARGFs. Results showed that ARGFs could be optimally coated by a layer of MnO_2_-based nanoparticles consisting of approximately 70% MnO_2_, 18% MnO, and 12% MnSiO_3_, when modified with an optimum OA to PP ratio of 10 for 24 h. The dissolution of ARGFs matrix in 4% and 10% NaOH solutions were distinctly delayed to 28 d, as a consequence of the introduction of the MnO_2_-based nanoparticle layer, compared with nontreated ARGF occurring at 3 d in 4% NaOH solution. For the optimally modified ARGFs, the mass loss was controlled to 1.76% and 2.91% after 90 d of corrosion in 4% and 10% NaOH solutions, and the retention of tensile strength was increased by approximately 25%. With respect to the increment in alkali-resistant performance, the modified ARGFs can be promising candidates for wide applications in alkaline cement-based products.

## 1. Introduction

Cement-based materials are widely used in building materials, such as precast panels and blocks for walls. Given the requirement for the toughness and strength of practical precast products in buildings, glass fibers are commonly employed to reinforce the cement-based materials [1,2], since they can exhibit better resistance to high temperatures and non-combustibility than most organic ones. However, common glass fibers are of relatively poor resistance to strong alkaline solution and degrade gradually in an alkaline environment [3,4]. Hence, the reinforcing effect on the toughness and strength of cement-based materials by glass fibers can be vastly hindered, which is due to the chemical alkali–silica reaction (ASR) between the alkali ions released from the hydration of the cement particles and the silica present inside glass fibers. Consequently, a diverse range of alkali-resistant glass fibers (ARGFs) has been designed accordingly. Such fibers can possess distinct resistance to attack from alkalinity and thus exhibit effective improvements on the toughness and strength of reinforced materials, depending on the physio-chemical property and surface modification process of glass fibers.

The ARGFs can generally be divided into two categories based on the manufacturing methods of the fiber materials. One common category is manufactured from formulating chemical compositions of a glass matrix with the optimum content of a rare earth or metal element [5,6]. The other one is mainly processed by coating the surface of fiber matrices with unique sizing agents [7,8] to improve the alkali resistance served in an alkaline environment. Depending on the chemical components and manufacturing process of the sizings, the ARGFs differ significantly in their alkali resistance performance.

With respect to the formulation of glass compositions, the incorporation of rare earth or metal elements can obviously increase the resistance to alkali attack in fibers [6,9], such as zirconia and titanium dioxide, etc. Dou et al. [9] investigated the effect of TiO_2_ on the alkali resistance of basalt fibers in 1 mol/L NaOH solution. The incorporation of 0.25% TiO_2_ by the mass of a basalt rock can form insoluble titanium hydroxide in the basalt fibers, which reduced the mass loss of the TiO_2_-basalt fibers at a corrosion age of 3 h by up to 50%. Lee et al. [10] utilized zirconia and refused coal ore for the fabrication of glass fibers. Their results showed that the tensile strength of glass fibers after alkali-attack test for 72 h was strengthened by 7% when zirconia and refused coal ore were applied compared with that of zirconia only. Lipatov et al. [11] reported that the basalt fibers, added with 5.1%, 8.1% zirconium oxide, and 1.0% lanthanum oxide, could exhibit comparable weight loss to zirconium-rich glass fibers (AR glass) after 3 h of corrosion in an alkaline solution mixed with 1 mol/L NaOH and 0.5 mol/L Na_2_CO_3_ by the ratio of 1:1. However, the utilization of rare earth or metal elements in manufacturing ARGFs led to a fairly high cost for ARGF-reinforced cement products, which hinders the wide use of such ARGFs in common cement-based materials.

The surface modification of glass fibers is taken to be a rational approach which could improve their alkali resistance. Diverse physical approaches, such as surface coating, sizing, and etching, and chemical strategies, such as oxidation, grafting, and chemical precipitation, have been put forward. Sayyar et al. [12] developed a low-cost weak-acid cation exchanger coating technique on glass fibers and found that modification with a weak-acid ion-exchanger consistently improved the flexural, shear, as well as compressive strength of the glass fiber-reinforced polymer bars serving in an alkaline solution under an accelerated aging test. Benmokrane et al. [13] investigated the effect of resin species on the alkaline resistance of glass fibers and found that glass fibers treated with a vinyl ester resin or sized with silanes had a better durability than other modifying agents. Kimm et al. [14] referred to a sanding process and 3-Amino-propyltriethoxysilane for modifying the surface of recycled glass fibers. Using this approach, they successfully enhanced the shearing strength of fiber-reinforced concrete and the bonding between the fibers and concrete matrix. Pan and Yan [15] investigated the effect of a carbon-fiber-reinforced polymer layer wrapped around glass fiber-reinforced polymer bars. The results revealed that the wrapping modification of the carbon fiber-reinforced polymer delayed the diffusion of the water and hydroxyl ions and, consequently, improved the shear strength retention as well as the degradation delay in terms of the mechanical properties.

Recent advances have also exhibited promise in nanomaterials in boosting the mechanical properties and bonding performance of fibers [16,17,18]. Signorini et al. [19] conducted a preliminary treatment of a nano silica coating on alkaline-resistant glass fabrics via sol-gel deposition and subsequently used this for a textile-reinforced mortar. It could be clearly seen that the tensile strength of the textile-reinforced mortar was doubly enhanced and the uncracked modulus was increased by 76.8% compared with the uncoated ones. They also applied micro and nano silica on alkaline-resistant glass fibers and woven hybrid carbon–alkaline-resistant glass fiber fabrics, which caused a notable enhancement in the tensile strength and deformability that could have originated from the surface tailoring effect as well as the pozzolanic effect between the reinforcing fibers and the mortar matrix. Naebe et al. [20] grafted amino-functionalized nanoclay on a carbon fiber surface and used it as a linkage between the fibers and epoxy matrix. Remarkable improvements, of approximately 60% in surface roughness and of 33% in interfacial shear strength, were gained, resulting in better interlocking and stress transfer between fibers and matrices. Other researchers [21,22] improved the mechanical performance of fibers by treating the surface of polypropylene fibers with acid-catalyzed sol-gel silica nano-coating and dip-coating E-glass fibers in TiO_2_ solutions to form surface coating. An awareness of the inferiority in nanomodification arose concurrently. Peled et al. [23] found that a nanosilica-modified coating did not facilitate the pullout strength and toughness of carbon multifilament yarn cement-based composites compared with that of micro silica. This could be ascribed to the extensive agglomeration of nanosilica as well as the segmentation formed in the drying procedures of silica coatings. Similar results were also observed by other researchers [24]. One of the effectual strategies for obviating the agglomeration effect of nanoparticles on fibers was in situ chemical growth, which intrinsically tailors the nucleation and growth of nanoparticles, bringing about the even and adjustable distribution of nanomaterials on fibers. However, limited studies have been reported focusing on synthesizing nanomaterials on glass fibers to improve their alkali resistance. There is a concern that the glass matrix of fibers can be efficiently protected by synthetic nanomaterials, regardless of the advantage of using nanomaterials to enhance the strength of cement-based materials.

The objective of the study herein is thus to improve the alkali resistance of glass fibers by modifying the fiber surface with nano materials, for instance, cement-based materials, that are resistant to high alkaline environments. Oleic acid and potassium permanganate were employed to synthesize manganese-dioxide-based nanomaterials on the surface of common commercial ARGF to further protect the fibers from alkali attack. The raw commercial ARGFs were treated with variable mass ratios of OA to PP (OA:PP) and variable modification times. The synthetic chemical compounds on ARGF after surface modification (ARGF-Mn) were analyzed using X-ray photoelectron spectroscopy (XPS) and obtaining scanning electron microscope (SEM) measurements. The alkali resistance performance of the ARGF-Mn was characterized by investigating the mass loss ratio and strength retention of the fiber materials in corrosive environments, prepared as 4% and 10% NaOH solutions. The outcomes of this study can provide new paths for the development of glass fibers with high alkali resistance.

## 2. Experimental Programmer

### 2.1. Materials

Sodium hydroxide (Sigma–Aldrich, St. Louis, MO, USA) and potassium permanganate (Laiyang Economic and Technological Development Zone Fine Chemicals, Laiyang, Shandong, China) of analytical grade were used to synthesize nanomaterials on the surface of glass fiber. Commercial common ARGF AR 5325 (Taishan Glass Fiber Co. Ltd., Tai’an, Shandong, China) of 1200 tex and 2.60 g/cm^3^ were employed as control fibers. Ordinary Portland cement P·O 42.5 (Shandong Sunnsy Cement Group Co. Ltd., Jinan, Shandong, China) corresponding to European CEM I 42.5 and standard quartz sands were used to measure the strength retention of glass fibers.

### 2.2. Surface Modification of Glass Fibers

The ARGFs with 8–10 g were mixed with oleic acid and 0.005 g/mL KMnO_4_ solution in a beaker. The OA:PP was set as 2, 6, 10, and 14, and the modification time was conducted as 12, 18, 24, and 30 h, respectively. Subsequently, the modified ARGF-Mn was washed with ethanol and distilled water, respectively, to remove unreacted residues, until the mixed solution became neutral, and was finally placed in a drying oven at 60 °C for 48 h.

### 2.3. Alkali Resistance Measurement

#### 2.3.1. Mass Loss Test

ARGF and ARGF-Mn of 12 pieces each (1 ± 0.0005 g) were placed in a vessel and then mixed with 4% and 10% NaOH solutions, respectively. After corrosion for 3, 7, 28, and 90 d, they were cleaned with distilled water until the pH value of the solution reached neutral. The cleaned glass fibers were immediately placed in a drying oven and dried at 60 °C to constant weight. The mass loss ratio of the glass fibers was determined using Equation (1), as follows:

(1) $$W=\frac{m_{0}-m_{1}}{m_{0}}\times 100\%$$

where W is the mass loss rate of glass fibers (%), $m_{0}$ is the initial mass of glass fibers (g), and $m_{1}$ is the mass of glass fibers after erosion (g).

#### 2.3.2. Strength Retention Test

Strength retention test complied with European standard EN 14649:2005 (E) [25]. Mixing design of mortar was set at water-to-cement ratio (w/c) of 0.43 and cement-to-sand ratio (c/s) of 3. In summary, a glass fiber strand 20 cm long was firstly laid across the center portion of the mold (30 mm × 10 mm × 10 mm). Adhesive tape assisted in holding the target fibers tightly. Then, epoxy resin was evenly coated on end portions of target fiber strands with central testing portion in 30 cm; it remained there and cured for certain duration. Subsequently, silicone sealant was employed at two ending points of testing portion of the fiber for impeding mortar impregnation. Thoroughly mixed mortar was poured into the aforementioned mold, tapped properly to exclude entrapped air voids, cured at 20 °C under relative humidity of 95% for 24 h, demolded and implemented with accelerating aging condition that immersed in distilled water at 80 °C for 4 d. Before the strength retention test, accelerating aging was terminated by cooling treatment at 20 °C for a quarter. Strength retention test was conducted by Material Testing System E42 at a loading rate of 1 mm/min with the strength retention gained by Equation (2) as follows:

(2) SR=P×ρ/T

where SR is the strength retention of fibers (MPa), P is the load measured at fracture (N), ρ represents density of fibers (g/cm^3^), and T is the linear density of fiber strand (1000 tex). The final strength retention was evenly acquired by twelve samples.

### 2.4. Characterization of Glass Fibers

The surface morphologies of ARGF and ARGF-Mn, as well as the morphology evolution of these fibers in alkali attack were characterized by scanning electron microscope (QUANTAFEG-250, Portland, OR, USA). Sputter coating of Au was conducted to enhance the conductivity of the samples. X-ray photoelectron spectrometer (ESCALAB 250, Themo Fisher SCIENTIFIC, Waltham, MA, USA) was used for surface composition analysis. Full scan together with high-resolution scan spectra were measured. Mn2p was analyzed by XPSPEAK software (version 4.1) with Gaussian-Lorentzian function. Peak fitting was employed based on subtraction of Shirley background. The optimal fit was finally gained by comprehensive effect of peak position, full width at half maximum, and peak intensity.

## 3. Results and Discussion

### 3.1. Surface Modification of Glass Fibers

#### 3.1.1. Morphology of Fibers by SEM

**Figure 1:**
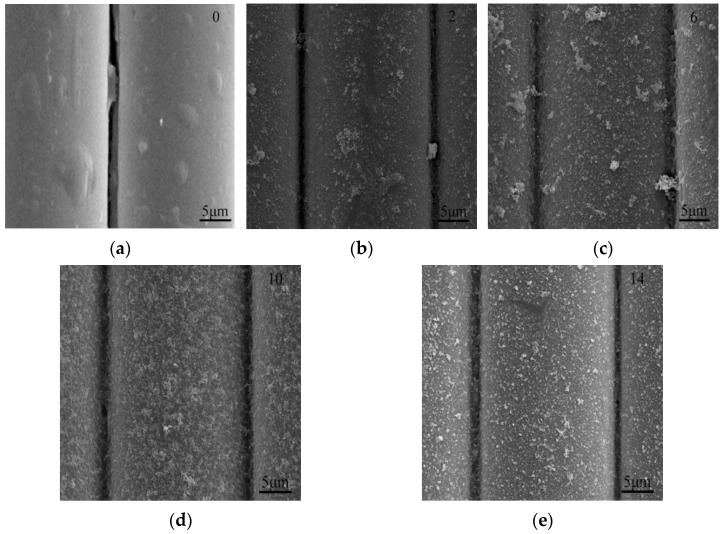
SEM images of alkali-resistant glass fiber modified with OA:PP ratio of (**a**) 0, (**b**) 2, (**c**) 6, (**d**) 10, and (**e**) 14, respectively. All modified fibers were treated for 24 h.

Figure 1 presents the surface morphology of alkali-resistant glass fibers modified with different ratios of OA: PP, after being treated for 24 h. The surface of ARGF-Mn was much rougher than that of the reference sample, given the nano-substances formed on the ARGF-Mn surface. The amount of nano-sized particles increased obviously and the distribution of such particles was more uniform with the rise in the OA:PP ratio. Furthermore, substantial nano-particles approximately perpendicular to the surface of ARGF-Mn were generated when the ratio of OA:PP was higher than 6. At a given OA:PP ratio of 10, the amount of nano-particle product was the largest and the surface of ARGF-Mn was fully and uniformly coated by these nano particles. Therefore, the optimal ratio of OA:PP was selected as 10 in this study.

**Figure 2:**
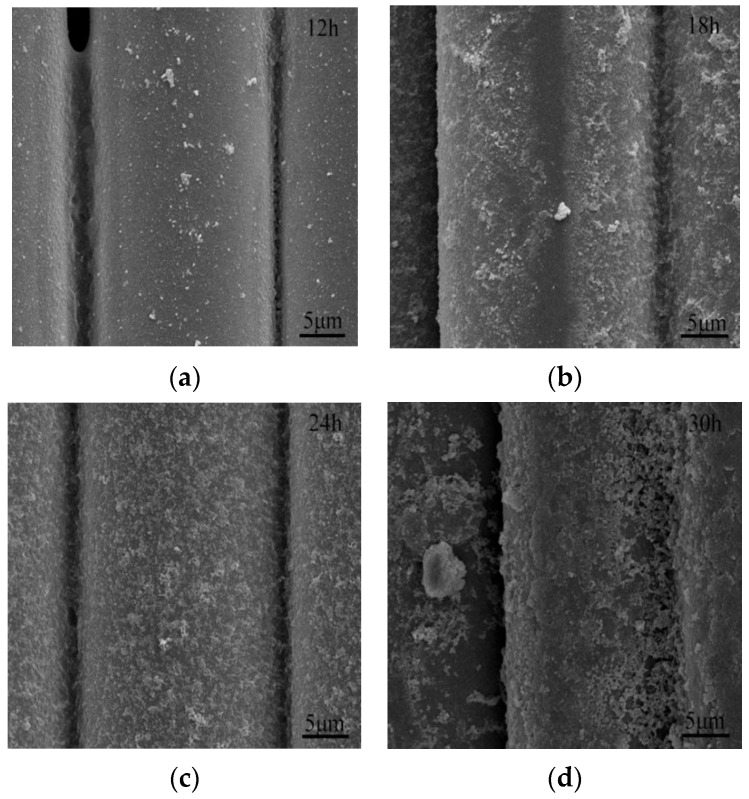
SEM images of alkali-resistant glass fibers modified with OA:PP ratio of 10 for (**a**) 12, (**b**) 18, (**c**) 24, and (**d**) 30 h, respectively.

The surface property of ARGF-Mn could also be affected by the duration of surface modification. Given the optimum ratio of OA:PP, the effect of modification time on morphology of ARGF-Mn surface was shown in Figure 2. The surface roughness of ARGF-Mn increased with the modification time by prolonging the chemical reaction between oleic acid and potassium permanganate from 12 to 24 h, due to the addition of nano-particles formed on the surface of ARGF. However, the surface roughness converted to decreasing when further modifying ARGF up to 30 h, given the densification of the nano-particle layer generated on the surface of ARGF-Mn. Based on the above SEM analysis, the modification time was optimally determined as 24 h, given the adapted OA:PP ratio of 10 in this study. With the modification approach built up above, the surface of alkali-resistant glass fibers could be well and uniformly covered with nano-particles, which were further analyzed to determine their chemical compositions.

#### 3.1.2. Synthetic Products by XPS

**Figure 3:**
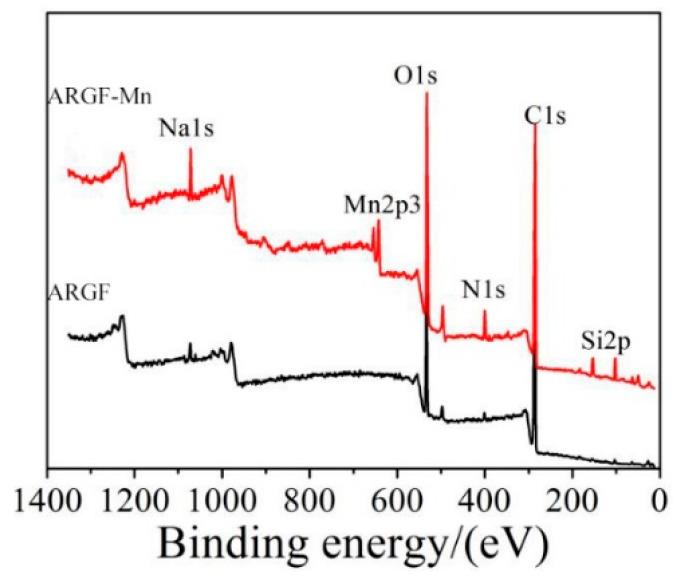
X-ray photoelectron spectra of ARGF-Mn and ARGF.

XPS measurements were conducted to determine the chemical substances formed on the surface of alkali-resistant glass fibers after modification. Figure 3 showed the XPS results of ARGF-Mn modified with an OA:PP ratio of 10 for 24 h and ARGF. The proportion of elements on the surface of ARGF-Mn and ARGF are presented in Table 1. The peaks of Si2p, N1s, O1s, and Na1s elements increased for the surface of ARGF-Mn, while that of C1s obviously reduced, compared with that of the ARGF surface. For example, the quantities of O1s element on the surface of glass fibers increased from 23.31% to 26.26% and that of C1s decreased from 72.37% to 58.86%, after surface modification was implemented. Furthermore, the XPS pattern of ARGF-Mn showed clear characteristic peaks of Mn2p3 element. The percentage of Mn2p3 element on the surface of ARGF-Mn was 2.22% by comparison with ARGF without Mn2p3. The increase in Mn2p3 and O1s percentage indicated that chemical compounds containing manganese ion existed on the surface of ARGF-Mn. The characteristic peak of Mn2p3 was further deconvolved and fitting analysis was then conducted in order to determine the chemical composition of the manganese-containing compounds on the surface of ARGF-Mn.

**Figure 4:**
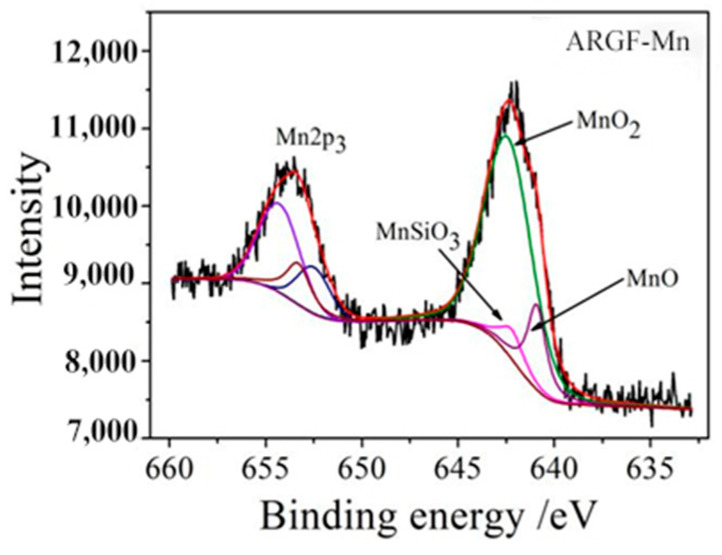
The Mn2p spectrum of ARGF-Mn surface with nano-MnO_2_.

Figure 4 displayed specific XPS peaks of manganese iron (Mn^x+^) for ARGF-Mn. Characteristic peaks of Mn2p_3/2_ and Mn2p_1/2_ elements were confirmed with respect to the binding energy located at around 642 eV and 653 eV, respectively. Such XPS characteristic peaks indicated the existence of chemical compounds containing Mn^4+^ and Mn^2+^ ions on the surface of ARGF-Mn. With respect to the Mn2p_3/2_ peak, it is shown that the binding energy of Mn^4+^ was 642.4 eV and that of Mn^2+^ differed in 642.2 eV and 641.0 eV [26]. The chemical compounds containing Mn^x+^ ions with binding energies of 642.4 eV, 642.2 eV, and 641 eV corresponded to MnO_2_, MnSiO_3_, and MnO, respectively. Hence, chemical products of MnO_2_, MnSiO_3_, and MnO were generated on ARGF-Mn. Further fitting of the Mn2p_1/2_ peak clearly showed the presence of the MnO_2_ product on the surface of the modified fibers. This can be ascribed to the reduction role of KMnO_4_ as strongly oxidant in its reaction with oleic acid, as follows:

The ions of Mn^7+^ from KMnO_4_ could be reduced to Mn^4+^, Mn^3+^, and even Mn^2+^ in the reaction between KMnO_4_ and oleic acid. The Mn^4+^ could precipitate in the stable form of MnO_2_, while the Mn^3+^ ions would undergo a dismutase reaction, by which they would be further reduced to Mn^2+^ and then oxidized into MnO_2_. The chemical reactions can be described by Equations (3) and (4) [27] accordingly:

(3) $$2{Mn}^{3+}+2H_{2}O={Mn}^{2+}+{MnO}_{2}↓+4H^{+}$$

(4) $$2{Mn}^{3+}\to {Mn}^{2+}+{Mn}^{4+}$$

Based on Equations (3) and (4), chemical compounds containing Mn^3+^ ions would not be formed on the surface of ARGF-Mn, whilst those composed of Mn^2+^ could be generated. This was consistent with the fitting results in Figure 4, showing that Mn^2+^ ions could exist in the form of MnO and MnSiO_3_. The presence of MnSiO_3_ substance can be attributed to the bonding of Mn^2+^ onto the surface of alkali-resistant glass fibers, in terms of -Mn-O-Si-, in which the element of silicon was provided by the glass fibers.

The atomic weight of Mn^4+^ and Mn^2+^ was approximately determined by calculating the peak area of their XPS fitting curves in Figure 4, as given in Table 2. The approximate proportions of MnO_2_, MnO, and MnSiO_3_ were then estimated as 70.58%, 17.81%, and 11.61%, respectively, given the molar mass of these chemical compounds. Hence, the major synthetic substance on ARGF-Mn was MnO_2_. Such synthetic chemical compounds could greatly affect the alkali-resistant properties of glass fibers that provide physical protection for the fiber surface.

### 3.2. Alkali-Resistant Performance of Glass Fibers

With synthetic MnO_2_-based chemical compounds adjusting the alkali-resistant properties of glass fibers, the alkali-resistant performance of glass fibers was studied by measuring the mass loss and strength retention of the fibers corroded in NaOH solution for variable durations of degradation.

#### 3.2.1. Mass Loss Ratio

**Figure 5:**
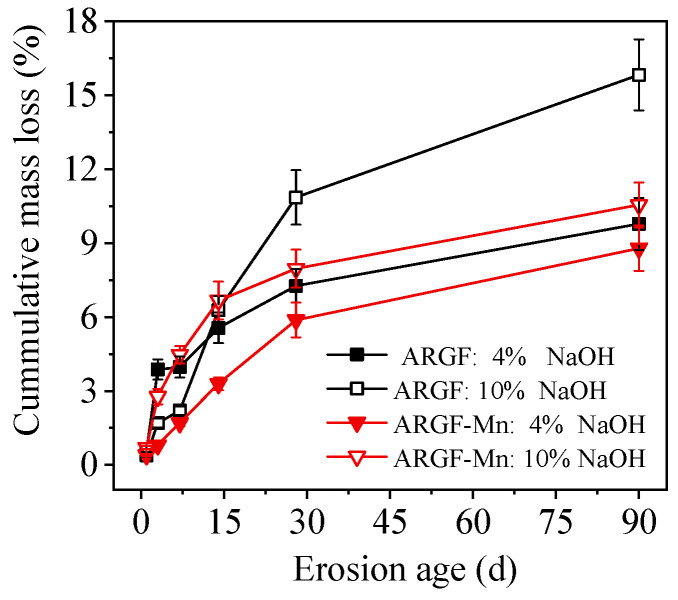
Evolution of mass loss ratio of ARGF-Mn and ARGF corroded in 4% and 10% NaOH solutions.

Figure 5 exhibits the mass loss ratio of ARGF-Mn and ARGF after corrosion treatment with 4% and 10% NaOH solutions for up to 90 d. The mass loss of alkali-resistant glass fibers rose with the extension of degradation due to the alkali-silica reaction between NaOH and silicate minerals of the fibers [3,4,27]. With alkaline ions present, i.e., OH^−^, silicate skeletons of glass fibers could be sensitively attacked by alkaline species, which led to the incontrovertible disconnection of silicate network. The connectivity of Si-O-Si was sharply diminished along with the increment of harsh alkaline ions transforming bridging oxygens into non-bridging ones, which accordingly accounted for the gradual strength corruption of glass fibers. Hence, the development of mass loss with time was obviously accelerated by enhanced NaOH concentration from 4% to 10% since it facilitated the alkali-silica reaction. For example, the mass loss ratio of ARGF corroded by 4% and 10% NaOH for 90 d was 2.51% and 4.97%, respectively. The corresponding chemical reaction could be expressed by Equation (5), as follows [3]:

(5) $$\equiv Si–O–Si\equiv +{OH}^{-}\to \equiv Si–OH+\equiv Si–O^{-}$$

The alkali-resistant performance of the glass fibers could be obviously improved by surface modification with an OA:PP ratio of 10 for 24 h. The mass loss ratio of ARGF-Mn corroded in 4% and 10% NaOH solution developed much slower with prolonged time than that of ARGF. The mass loss ratio of ARGF-Mn after 90 d of corrosion with 4% and 10% NaOH solution was dropped to 1.76% and 2.91%, respectively.

**Figure 6:**
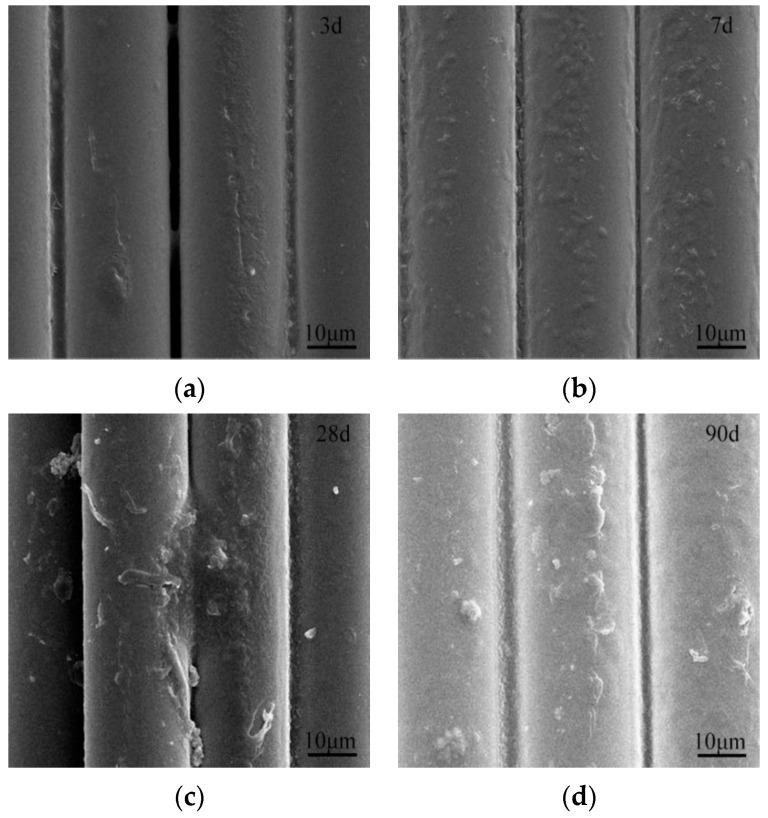
SEM images of ARGF corroded by 4% NaOH solution for (**a**) 3, (**b**) 7, (**c**) 28, and (**d**) 90 d.

**Figure 7:**
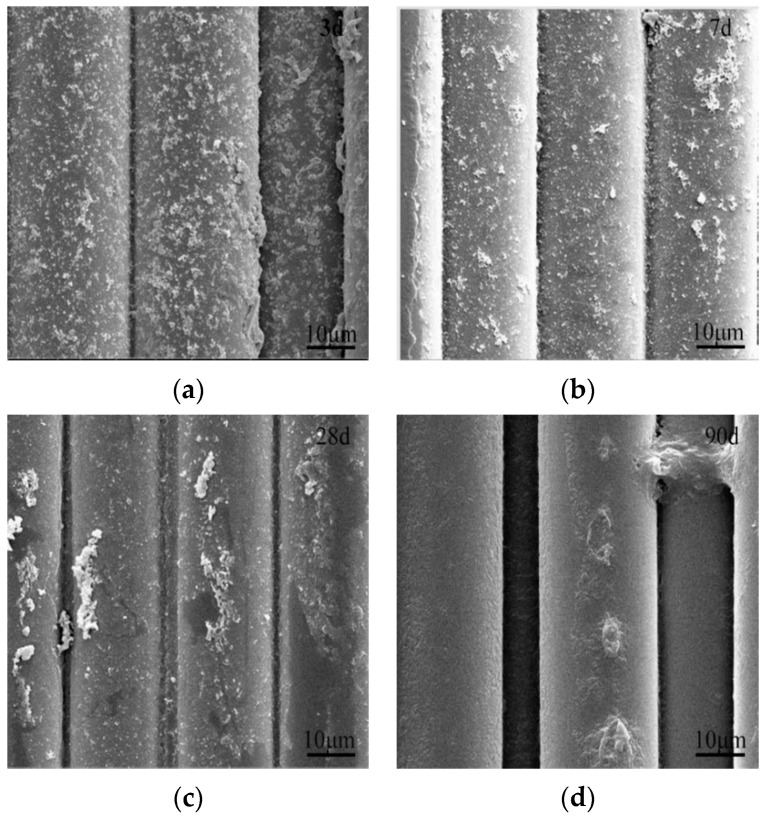
SEM images of ARGF-Mn corroded by 4% NaOH solution for (**a**) 3, (**b**) 7, (**c**) 28, and (**d**) 90 d.

Figure 6 and Figure 7 performed microscopic morphologies of ARGF and ARGF-Mn eroded by 4% NaOH solution for 3, 7, 28, and 90 d. The smoothness of ARGF surface decreased obviously owing to the chemical attack of NaOH on the surface of the fibers. The surface of ARGF was slightly pitted at 3 d of corrosion, then the overall surface was gradually dissolved from 7 d to 90 d. For ARGF-Mn modified with an OA:PP of 10 for 24 h, the surface of the fiber matrices were remarkably protected from alkali corrosion, as presented in Figure 7. This was ascribed to the coating layer of MnO_2_-based nanoparticles formed on the surface of ARGF-Mn, which can provide physical protection for the modified fibers from the NaOH-silica reaction. The protective nanoparticles decreased gradually during the corrosion from 3 d to 28 d by 4% NaOH solution and diminished at 90 d.

**Figure 8:**
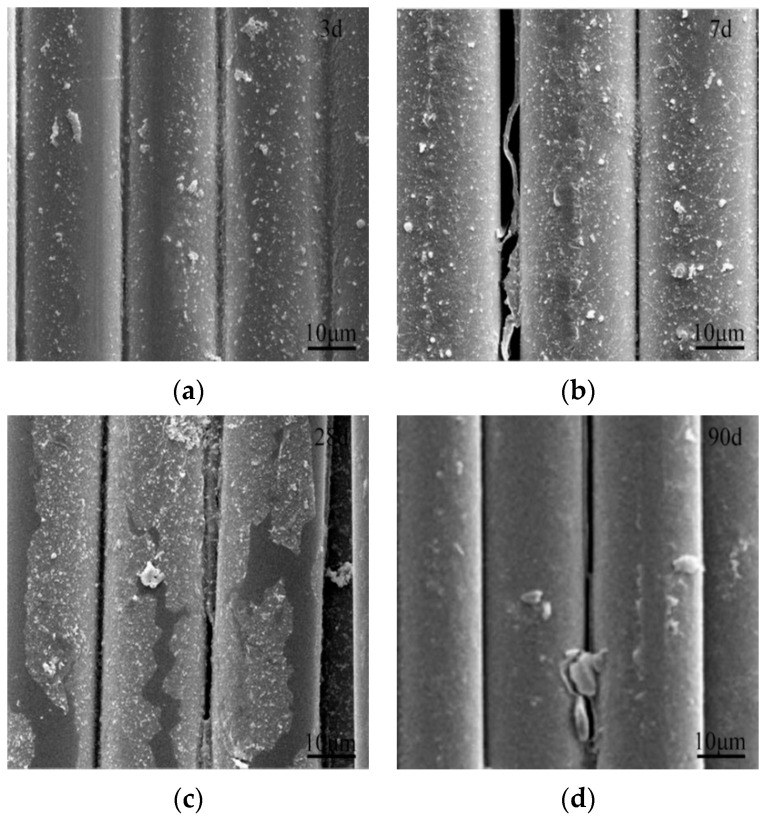
SEM images of ARGF-Mn corroded by 10% NaOH solution for (**a**) 3, (**b**) 7, (**c**) 28, and (**d**) 90 d.

The degradation process of MnO_2_-based nanoparticles obviously accelerated with the increasing NaOH concentration from 4% to 10%. Substantial MnO_2_-based nanoparticles were dissolved at 3 d of corrosion in 10% NaOH solution, as shown in Figure 8. Such intensive dissolution of the nanoparticles was delayed from 7 to 28 d of corrosion in 4% NaOH solution. Furthermore, large pieces of the protective layer of MnO_2_-based nanoparticles were peeled off from the surface of ARGF-Mn after 28 d of corrosion with 10% NaOH solution. For ARGF-Mn corroded in 4% NaOH solution, only a small portion of the protective layer was destroyed at 28 d, as shown in Figure 7. The MnO_2_-based nanoparticles formed on the surface of ARGF-Mn were fully degraded after 90 d of corrosion in 4% and 10% NaOH solution. Still, the surface of ARGF-Mn at 90 d was less rough than that of ARGF, resulting from the enhancement of alkali resistance of ARGF-Mn due to the protection of MnO_2_-based nanoparticle layer.

#### 3.2.2. Strength Retention

**Figure 9:**
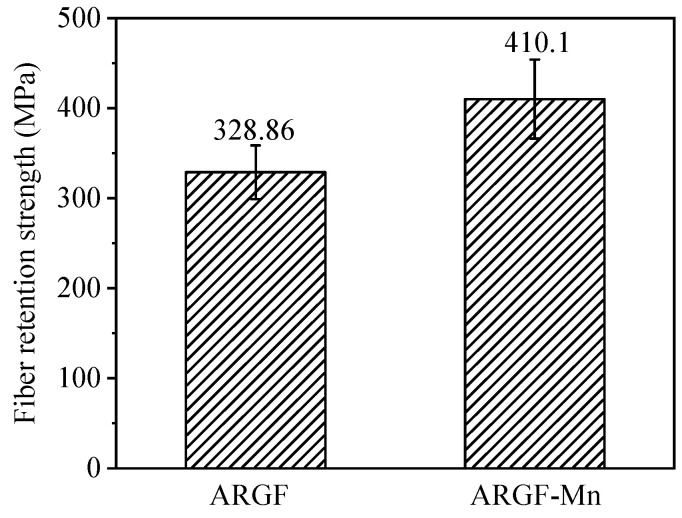
Strength retention of ARGF and ARGF-Mn modified with the optimal OA:PP ratio of 10 for 24 h.

Figure 9 illustrates the retention strength of ARGF and ARGF-Mn with optimal surface modification in Section 3.1 under high-alkaline cement-based environments. The average retention strength of ARGF and ARGF-Mn was 328.86 MPa and 410.10 MPa, respectively. Compared to ARGF, the average retention strength of ARGF-Mn was improved by 24.7%. This could be attributed to the nano-MnO_2_ based coatings on the surface of ARGF-Mn. Such a coating could resist corrosion, in alkali environments, from the hydration of cement particles and thus delay the corrosion of alkalis to glass fiber matrices. As a result, the strength of ARGF-Mn could be retained to a great extent before the nano-MnO_2_ was exhausted thoroughly. By contrast, ARGF was completely exposed to harsh alkali environments and suffered from aggressive alkaline attack. Thus, the Si-O network of ARGF was more vulnerable to be broken, leading to the rapid degradation of the strength of ARGF.

## 4. Conclusions

In this study, nanoparticles mainly consisting of MnO_2_ were successfully constructed on the surface of ARGF by employing oleic acid and potassium permanganate as reactants, in order to further defend against alkali attacks on ARGF in alkaline environments. The mass loss ratio and strength retention of the modified ARGF-Mn exposed to NaOH solution were investigated for evaluating the effect of synthetic nanoparticles on the alkali resistance of the fibers. Based on the aforementioned, the following conclusions can be drawn:

The surface of ARGF was optimally coated with nanoparticles when modified with an OA:PP ratio of 10 for 24 h, with a broad and uniform distribution of the nanoparticles on ARGF-Mn surface successfully acquired. The synthetic compound of MnO_2_ accounted for more than 70% of the nanoparticles, while MnO and MnSiO_3_ took up approximately 18% and 12%, respectively.

The coating layer of MnO_2_-based nanoparticles delayed the obvious dissolution of the ARGF matrix in 4% and 10% NaOH solutions to the corrosion age of 28 d, while nonmodified ARGF was obviously pitted at only 3 d of corrosion in 4% NaOH solution. After 90 d of corrosion in 4% and 10% NaOH solutions, the mass loss of the optimal ARGF-Mn was significantly reduced to 1.76% and 2.91%, compared with that of ARGF of 2.51% and 4.97%, respectively.

With the optimum modification of the fiber surface, the retention of tensile strength of ARGF-Mn was approximately 25% higher than that of ARGF, illuminating the fact that the MnO_2_-based coating layer established could effectively resist attacks of alkalinity from the hydration of cement particles on an ARGF matrix.

## Figures and Tables

**Table 1:** Proportion of elements on the surface of ARGF-Mn and ARGF.

Fiber Type	Elemental Percentage (%)					
	Si2p	C1s	N1s	O1s	Mn2p3	Na1s
ARGF	1.33	72.37	1.94	23.31	0	0.96
ARGF-Mn	5.33	58.86	4.76	26.26	2.22	2.01

**Table 2:** Binding energy of Mn^x+^ and peak area in Mn2p_3/2_ and Mn2p_1/3_.

Binding energy (eV)	640.9	642.2	642.4	652.5	653.3	654.3	642.2
Peak area (a.u.)	1876.86	846.42	9500.52	1289.53	1218.18	3049.04	846.42

## Data Availability

The data are contained within the article. Additional data are available upon request from the corresponding authors.
